# Plasma Amyloid Beta and Tau Levels Are Predictors of Post-stroke Cognitive Impairment: A Longitudinal Study

**DOI:** 10.3389/fneur.2019.00715

**Published:** 2019-07-02

**Authors:** Nai-Fang Chi, Shu-Ping Chao, Li-Kai Huang, Lung Chan, Yih-Ru Chen, Hung-Yi Chiou, Chaur-Jong Hu

**Affiliations:** ^1^Department of Neurology, School of Medicine, College of Medicine, Taipei Medical University, Taipei, Taiwan; ^2^Department of Neurology, Stroke Center, Shuang Ho Hospital, Taipei Medical University, New Taipei, Taiwan; ^3^Department of Neurology, Faculty of Medicine, National Yang-Ming University School of Medicine, Taipei, Taiwan; ^4^Department of Neurology, Neurological Institute, Taipei Veterans General Hospital, Taipei, Taiwan; ^5^School of Public Health, College of Public Health, Taipei Medical University, Taipei, Taiwan; ^6^Graduate Institute of Neural Regenerative Medicine, College of Medical Science and Technology, Taipei Medical University, Taipei, Taiwan; ^7^Taipei Neuroscience Institute, Taipei Medical University, Taipei, Taiwan

**Keywords:** tau, Amyloid (A) 42, post-stroke cognitive impairment, vascular cognition impairment, Montreal Cognitive Assessment (MoCA)

## Abstract

**Objectives:** Post-stroke cognitive impairment (PSCI) is a common disease that may occur within 3 months after a stroke or even later. However, the mechanism of PSCI development is unclear. The present study investigated whether the levels of plasma amyloid beta-42 (Aβ42) and tau are associated with the onset of PSCI.

**Methods:** Fifty-five patients admitted within 7 days of acute ischemic stroke were enrolled and followed up for 1 year. Montreal Cognitive Assessment (MoCA) was administered at 3 months and 1 year, and plasma Aβ42 and tau levels were determined using an ultrasensitive immunoassay (immunomagnetic reduction) within 7 days of the stroke event and 3 months later.

**Results:** In this study, 13 of 55 patients developed PSCI (MoCA score <23) at 3 months. Seven patients with PSCI at 3 months recovered to a cognitively normal state at 1 year, whereas seven cognitively normal patients developed PSCI at 1 year. The patients with PSCI at 1 year had a higher incidence of cognitive function deterioration between 3 months and 1 year compared with those without PSCI at 1 year. Plasma Aβ42 and tau levels at 3 months were lower in the patients with PSCI at 1 year than in those without PSCI (Aβ42: 15.1 vs. 17.2 pg/mL, *P* = 0.013; tau: 16.7 vs. 19.9 pg/mL, *P* = 0.018). Low education levels and pre-existing white matter disease were the most significant predictors of PSCI at 3 months, and poor cognitive performance at 3 months and low plasma Aβ42 and tau levels at 3 months were the most significant predictors of PSCI at 1 year.

**Conclusion:** The pathogenesis of PSCI is complex and changes with time. Ischemia-induced Aβ42/tau pathology might be involved in PSCI development.

## Introduction

Post-stroke cognitive impairment (PSCI) can occur immediately after a stroke or after a certain period. PSCI occurrence after a certain period is common, but it is often overlooked. Post-stroke dementia occurs in 7% of patients 1 year after a stroke (1); however, its incidence could be as high as 52% at 6 months after a stroke if mild PSCI is included in the diagnosis (2). PSCI is associated with impairment of activities of daily living, poor quality of life, and increased burden of care (3, 4). In addition, compelling evidence indicates that accelerated, continuous cognitive decline may occur following acute stroke events, even without clinically recurrent stroke occurrence (5). Consequently, in addition to the standard healthcare for stroke management, it is essential to detect and prevent PSCI. Age, hypertension, diabetes, and low education levels have been reported to be associated with PSCI (6). The roles of neuroinflammation and amyloid-related pathology in PSCI development have also been highlighted (7). However, the pathogenesis of late-onset PSCI remains unclear. In addition, precise biomarkers that could facilitate the prediction of PSCI following acute stroke events are lacking.

Cerebral ischemia triggers amyloid beta-42 (Aβ42) deposition, alteration of tau phosphorylation, and neuroinflammation (7–9), which are also the core pathologies of Alzheimer's disease (AD). Therefore, in addition to damaging the brain directly, ischemia may induce neurodegenerative processes that lead to PSCI development. The levels of Aβ42 and tau in the cerebrospinal fluid (CSF) correlate well with the brain Aβ42 burden assessed using position emission tomography (PET) (10, 11). The Aβ42 burden on PET images is the gold standard maker for AD diagnosis; however, it is not widely applied in clinical practice. The Aβ42 level in the plasma correlates with the Aβ42 level in the CSF, and the Aβ42 levels in the plasma are lower in patients with AD than in patients with mild cognitive impairment or controls (12, 13). In addition, the plasma Aβ42 levels and Aβ42/Aβ40 ratios could reveal AD pathological change in PET among cognitively normal individuals with subjective cognitive decline (12). In addition, the levels of plasma tau are higher in patients with AD than in controls (14, 15). The Aβ42, Aβ40, and tau levels in peripheral blood can be quantified using ultrasensitive immunoassays such as single-molecule assay (SIMOA) or immunomagnetic reduction (IMR) assay. Aβ42 and tau plasma measurements are more practical than PET and CSF assessments in clinical settings. Therefore, plasma Aβ42 and tau are potential biomarkers of neurodegenerative diseases, including PSCI. The association between plasma Aβ42 levels and cognitive function was investigated in a cross-sectional study of stroke (16). A longitudinal study would clarify the temporal relationship between cognitive function deterioration and the plasma biomarkers of Aβ42 and tau.

The roles of plasma Aβ42 and tau in PSCI development are still unclear. We hypothesized that the levels of plasma Aβ42 and tau may predict the onset of PSCI. In the present study, we investigated whether plasma Aβ42 and tau levels at 3 months after an ischemic stroke are associated with the onset of PSCI at 3 months and 1 year.

## Methods

### Participants

The Institutional Review Board of Taipei Medical University approved the present study. Adult patients (≥20 years old) who were admitted to Taipei Medical University-Shuang Ho Hospital within 7 days of acute ischemic stroke were consecutively screened for eligibility in the present study. The exclusion criteria were as follows: (1) Patients with known cognitive impairment or a neurodegenerative disease that impaired regular daily activities before a stroke, (2) patients with large infarcts (≥1/3 middle cerebral artery territory) that caused immediate consciousness or cognitive impairment after a stroke, (3) patients with strategic infarcts involving the paramedian thalamus, hippocampus, or medial frontal cortex with a high risk of cognitive impairment, and (4) patients with severe language or physical disability that impeded neuropsychological testing. Each patient was assessed within 7 days of a stroke at admission and was followed up at 3 months and 1 year at the outpatient clinic. Sixty patients were enrolled into the study, and written informed consent was obtained from all patients or their legal guardians.

### Clinical Characteristics

The patients' stroke severity was assessed at admission, 3 months, and 1 year using the National Institute of Health Stroke Scale (NIHSS), and the patients' cognitive functions were assessed using the Montreal Cognitive Assessment (MoCA) screening instrument (17) at 3 months and 1 year. A trained research assistant blinded to the patients' plasma biomarker data conducted assessments. The MoCA score was adjusted based on the patients' education levels. The patients who could not complete the MoCA test at 3 months due to physical disability (e.g., dominant hand weakness and unable to complete the drawing tasks) were excluded at 3 months (*n* = 5). Data of 55 patients were included in the final analysis. The patients with a MoCA score of <23 were considered as having cognitive impairment (18).

Brain magnetic resonance images were obtained once at admission, including T1- and T2-weighted images, T2 fluid-attenuated inversion recovery (T2 FLAIR) images, diffusion-weighted images (DWI), apparent diffusion coefficient maps, and time-of-flight magnetic resonance angiograms. Carotid Doppler ultrasonographs and electric cardiograms were also obtained once during admission. The lesion volume on DWI was calculated manually, pre-existing white matter hyperintensities in T2 FLAIR images were assessed using the Fazekas scale (19), and the etiologic subtype of stroke was classified based on the TOAST study (20) by an experienced neurologist blinded to the patients' outcomes.

### Plasma Amyloid Beta and Tau Protein Analysis

The patients provided a 10-mL non-fasting venous blood sample within 7 days of the stroke event and 3 months later. The blood samples were centrifuged at 1,500 × g for 15 min and stored at −80°C. The blood samples were sent to MagQu Co. Ltd. (New Taipei City, Taiwan) for IMR assays, with blinding applied for the patients' identity and clinical information.

The technical details of IMR assays are described in previous studies (21, 22). In brief, IMR reagents consisting of Fe_3_O_4_ nanoparticles coated with Aβ42, Aβ40, or tau antibodies (MF-AB2-0060, MF-AB0-0060, and MF-TAU-0060 respectively, MagQu) were used to capture the antigens in the plasma. The antigen-bound nanoparticles became larger, clustered, and their movements were less responsive to an external magnetic field than the unbound ones. Reduction in magnetic susceptibility was measured using a magnetosusceptometer (XacPro-S, MagQu). The percentage reduction in magnetic susceptibility was referred to as IMR. The plasma antigen levels were determined using a logistic function of the concentration-dependent IMR signal. Aβ40 levels were measured only at 3 months after the stroke event. Each reported result of Aβ or tau in a patient was the average of duplicate measurements that had a coefficient of variation <15%.

### Statistical Analysis

Data normality was determined using the Shapiro–Wilk test. Normally distributed data are expressed as means ± standard deviations, whereas non-normally distributed data are expressed as medians with inter-quartile ranges (IQRs). Demographic and laboratory data were compared between the patients with PSCI (MoCA score <23) and those without PSCI (MoCA score ≥ 23) using the *t*-test, Mann–Whitney *U*-test, or χ^2^ test, as applicable. The MoCA score and laboratory data at different time points were compared using the Wilcoxon signed-rank test. Correlations between variables were verified using the Spearman rank correlation coefficient. Univariate logistic regression was used to estimate the odds ratios of PSCI onset based on clinical characteristics and laboratory data. All variables were tested for multicollinearity (variance inflation factor ≥ 10). Multivariate logistic regression using automatic forward variable selection (enter if *P* < 0.10) was conducted to build a model including significant variables associated with PSCI. A *P*-value of < 0.05 was considered statistically significant. Statistical data were analyzed using MedCalc v18 (MedCalc Software bvba, Ostend, Belgium).

## Results

The clinical characteristics of the patients with PSCI at 3 months (*n* = 13) and without PSCI at 3 months (*n* = 42) are summarized in Table 1. Most patients had mild stroke severity (median NIHSS score = 4 and median lesion volume = 0.5 cm^3^ in each group at admission) and small-vessel disease, which was consistent with our study inclusion and exclusion criteria. Education levels in the patients with PSCI at 3 months were significantly lower than those in the patients without PSCI at 3 months, whereas the scores of the Fazekas scale in the patients with PSCI at 3 months were significantly higher than those in the patients without PSCI at 3 months. Age, sex, stroke lesion side, stroke etiologic subtype, vascular comorbidities, NIHSS score within 7 days, NIHSS score at 3 months, hemoglobin A1c (HbA1c), lesion volume on DWI, plasma Aβ42, and tau levels within 7 days, and Aβ42, Aβ40, and tau levels at 3 months were not different between the patients with and without PSCI at 3 months.

**Table 1:** Characteristics of the participants categorized based on PSCI development at 3 months.

	**PSCI (–) at 3 months (*n* = 42)**	**PSCI (+) at 3 months (*n* = 13)**	***P*-value**
Age, median (IQR)	59 (50 - 65)	62 (61–68)	0.122
Male sex	33 (79%)	12 (92%)	0.266
Education level	12 (9–16) years	9 (6–12) years	0.034*
Hypertension	32 (76%)	9 (69%)	0.618
Diabetes mellitus	14 (33%)	8 (62%)	0.072
Hemoglobin A1c ≤ 7 days, median (IQR)	5.9 (5.5–7.7)%	6.3 (6.1–7.6)%	0.212
Hyperlipidemia	29 (69%)	9 (69%)	0.990
NIHSS ≤ 7 days, median (IQR)	4 (2–5)	4 (1–4)	0.603
Stroke etiology			0.368
Large-artery atherosclerosis	3 (7%)	3 (23%)	
Small-vessel disease	32 (76%)	9 (69%)	
Cardioembolism	5 (12%)	1 (8%)	
Undetermined etiology	2 (5%)	0 (0%)	
DWI lesion volume, median (IQR)	0.5 (0.3–1.1) cm^3^	0.5 (0.1–10.2) cm^3^	0.890
DWI lesion on the left side	21 (51%)	6 (50%)	0.941
Fazekas scale, periventricular + deep white matter, median (IQR)	1 (0–2)	2 (1–3)	0.006*
Plasma biomarkers, median (IQR)			
Amyloid beta-42 ≤ 7 days	16.0 (14.4–17.9) pg/mL	17.1 (15.6–17.3) pg/mL	0.583
Tau ≤ 7 days	18.2 (14.4–33.0) pg/mL	22.0 (17.1−29.3) pg/mL	0.303
Amyloid beta-42 at 3 months	16.5 (15.3–18.5) pg/mL	15.1 (14.5–18.3) pg/mL	0.352
Amyloid beta-40 at 3 months	49.7 (44.3–54.0) pg/mL	50.9 (49.2–54.1) pg/mL	0.362
Amyloid beta-42/40 ratio at 3 months	32 (28–42)%	29 (28–36)%	0.247
Tau at 3 months	19.1 (14.4–26.7) pg/mL	17.4 (14.9–29.0) pg/mL	0.937

* *P < 0.05, DWI, diffusion-weighted image; IQR, interquartile range; NIHSS, National Institute of Health Stroke Scale; PSCI, post-stroke cognitive impairment*.

**Figure 1:**
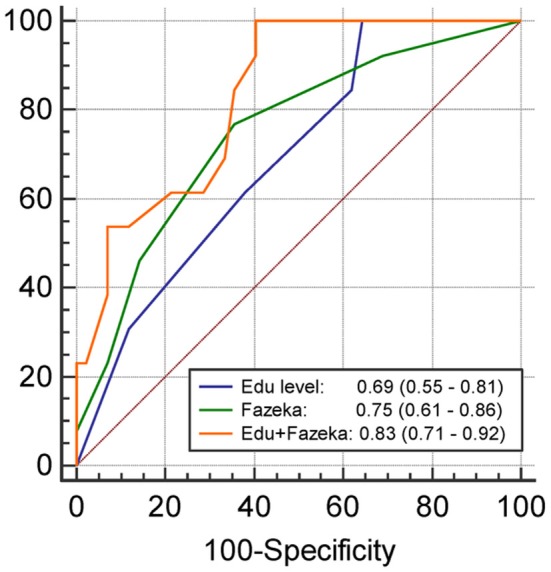
The receiver operating characteristics curve and the area under curves (95% confidence interval) of the variables predicting PSCI at 3 months. Edu, Education level (year); Fazeka, The score of Fazekas scale.

The results of univariate and multivariate logistic regression analyses of variables predicting PSCI at 3 months are summarized in Table 2. In univariate analysis, the education level and Fazekas scale score were significant predictors, whereas age, diabetes mellitus, and lesion volume on DWI were predictors with borderline significance (*P* = 0.080, 0.077, and 0.059, respectively). The NIHSS score within 7 days or at 3 months, hypertension, hyperlipidemia, HbA1c, stroke lesion side, plasma Aβ42, and tau levels within 7 days, and plasma Aβ42, Aβ40, and tau levels at 3 months were not predictors of PSCI at 3 months. In multivariate analysis, only the education level and Fazekas scale score were included in the automatic forward variable selection model with an acceptable model fit (Nagelkerke *R*^2^ = 0.37, Hosmer–Lemeshow test *P* = 0.17). Therefore, the education level and pre-existing white matter disease (Fazekas scale score) were the most significant predictors of PSCI at 3 months. The receiver operating characteristic (ROC) curves of the education level, Fazekas scale score, and multivariate model for predicting PSCI at 3 months are displayed in Figure 1.

**Table 2:** Univariate and multivariate logistic regression analyses of PSCI at 3 months.

**Characteristics (*n* = 55)**	**Crude OR (95% CI)**	***P*-value**	**Adjusted OR^†^ (95% CI)**	***P*-value**
Age	1.07 (0.99–1.16)	0.080		
Male sex	3.27 (0.37–28.64)	0.284		
Education level (years)	0.82 (0.67–0.99)	0.036*	0.76 (0.60–0.96)	0.021*
Hypertension	0.70 (0.18–2.78)	0.616		
Diabetes mellitus	3.20 (0.88–11.61)	0.077		
Hemoglobin A1c (%) ≤ 7 days	1.09 (0.77–1.52)	0.635		
Hyperlipidemia	1.01 (0.26–3.88)	0.990		
NIHSS ≤ 7 days	0.93 (0.70–1.23)	0.607		
DWI lesion volume (cm^3^)	1.11 (1.00–1.24)	0.059		
DWI lesion on the left side	0.95 (0.26–3.45)	0.941		
Fazekas scale (periventricular + deep white matter)	1.99 (1.19–3.33)	0.009*	2.31 (1.29–4.15)	0.005*
Plasma biomarkers				
Amyloid beta-42 (pg/mL) ≤ 7 days	0.97 (0.74–1.26)	0.803		
Tau (pg/mL) ≤ 7 days	1.01 (0.95–1.08)	0.659		
Amyloid beta-42 (pg/mL) at 3 months	0.88 (0.67–1.15)	0.334		
Amyloid beta-40 (pg/mL) at 3 months	1.04 (0.94–1.15)	0.412		
Amyloid beta-42/40 ratio (%) at 3 months	0.96 (0.89–1.03)	0.259		
Tau (pg/mL) at 3 months	1.00 (0.92–1.08)	0.951		

* *P < 0.05*,

† *Education level and Fazekas scale score were included in the automatic forward variable selection model, DWI, diffusion-weighted image; NIHSS, National Institute of Health Stroke Scale; PSCI, post-stroke cognitive impairment*.

**Figure 2:**
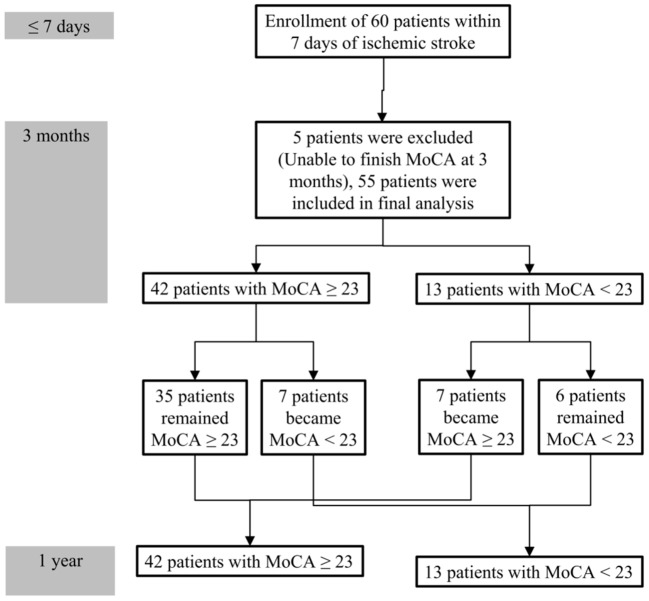
Flowchart of patient enrollment and cognitive function changes during the 1-year follow-up.

During follow-up, the median MoCA scores of all participants were not different between 3 months and 1 year (25 vs. 25, *P* = 0.314). However, 7 of 42 patients without PSCI at 3 months exhibited cognitive function deterioration, which progressed to PSCI at 1 year. Conversely, 7 of 13 patients with PSCI at 3 months exhibited improvements in cognitive function, and PSCI was reversed at 1 year. A comparison of MoCA scores (positive values represented improvements, and negative values represented deterioration) between the patients with and without PSCI at 1 year is presented in Table 3. The incidence of cognitive function deterioration (MoCA score decreased by 2 points or more) was significantly higher in the patients with PSCI at 1 year than in the patients without PSCI at 1 year. In addition, MoCA scores at 3 months were significantly lower in the patients with PSCI at 1 year than in the patients without PSCI at 1 year. Figure 2 presents a flowchart of patient enrollment and changes in cognitive function.

**Table 3:** Characteristics of MoCA categorized based on PSCI development at 1 year.

	**PSCI (–) at 1 year (*n* = 42)**	**PSCI (+) at 1 year (*n* = 13)**	***P*-value**
MoCA score change (3 months to 1 year)			< 0.001*
≤−2	3 (7%)	10 (77%)	
−1 to +1	23 (55%)	0 (0%)	
≥+2	16 (38%)	3 (23%)	
Median (IQR)	1 (−1 to 3)	−3 (−4 to −0.5)	0.003*
MoCA score at 3 months, median (IQR)	26 (23–28)	23 (16–25)	0.002*

* *P < 0.05, IQR, interquartile range; MoCA, Montreal Cognitive Assessment; PSCI, post-stroke cognitive impairment*.

The clinical characteristics of the patients with PSCI at 1 year (*n* = 13) and without PSCI at 1 year (*n* = 42) are summarized in Table 4. Plasma Aβ42 and tau levels at 3 months as well as the Aβ42/40 ratio at 3 months in the patients with PSCI at 1 year were significantly lower than those in the patients without PSCI at 1 year. Age, sex, education level, stroke lesion side, stroke etiologic subtype, vascular comorbidities, HbA1c, NIHSS score within 7 days, NIHSS score at 3 months, NIHSS score at 1 year, lesion volume on DWI, and plasma Aβ42 and tau levels within 7 days were not different between the patients with and without PSCI at 1 year.

**Table 4:** Characteristics of the participants categorized based on PSCI development at 1 year.

	**PSCI (–) at 1 year (*n* = 42)**	**PSCI (+) at 1 year (*n* = 13)**	***P*-value**
Age, median (IQR)	60 (53–64)	65 (60–68)	0.100
Male Sex	34 (81%)	11 (85%)	0.767
Education level	12 (9–16) years	9 (6–12) years	0.090
Hypertension	32 (76%)	9 (69%)	0.618
Diabetes mellitus	15 (36%)	7 (54%)	0.248
Hemoglobin A1c ≤ 7 days, median (IQR)	6.2 (5.6–7.7)%	5.8 (5.7–7.5)%	0.984
Hyperlipidemia	28 (67%)	10 (77%)	0.488
NIHSS ≤ 7 days, median (IQR)	4 (2–5)	4 (2–4)	0.857
NIHSS at 3 months, median (IQR)	1 (0–2)	2 (0–3)	0.140
NIHSS at 1 year, median (IQR)	0 (0–1)	1 (0–2)	0.276
Stroke etiology			0.727
Large-artery atherosclerosis	4 (10%)	2 (15%)	
Small-vessel disease	32 (76%)	9 (69%)	
Cardioembolism	5 (12%)	1 (8%)	
Undetermined etiology	1 (2%)	1 (8%)	
DWI lesion volume, median (IQR)	0.5 (0.3–1.0) cm^3^	0.5 (0.1–10.2) cm^3^	0.729
DWI lesion on the left side	20 (49%)	7 (58%)	0.564
Fazekas scale, periventricular + deep white matter, median (IQR)	1 (0–2)	1 (1−2)	0.992
Plasma biomarkers, median (IQR)			
Amyloid beta-42 ≤ 7 days	16.0 (14.4–17.8) pg/mL	16.4 (15.4–17.7) pg/mL	0.478
Tau ≤ 7 days	18.6 (14.4–33.1) pg/mL	18.9 (16.2–21.6) pg/mL	0.927
Amyloid beta-42 at 3 months	17.2 (15.3–19.2) pg/mL	15.1 (14.6–16.5) pg/mL	0.013*
Amyloid beta-40 at 3 months	49.5 (44.3–53.3) pg/mL	51.1 (49.1–55.5) pg/mL	0.087
Amyloid beta-42/40 ratio at 3 months	32 (29–44)%	29 (28–30)%	0.010*
Tau at 3 months	19.9 (15.0–28.9) pg/mL	16.7 (12.8–18.9) pg/mL	0.018*

* *P < 0.05, DWI, diffusion-weighted image; IQR, interquartile range; NIHSS, National Institute of Health Stroke Scale; PSCI, post-stroke cognitive impairment*.

**Figure 3:**
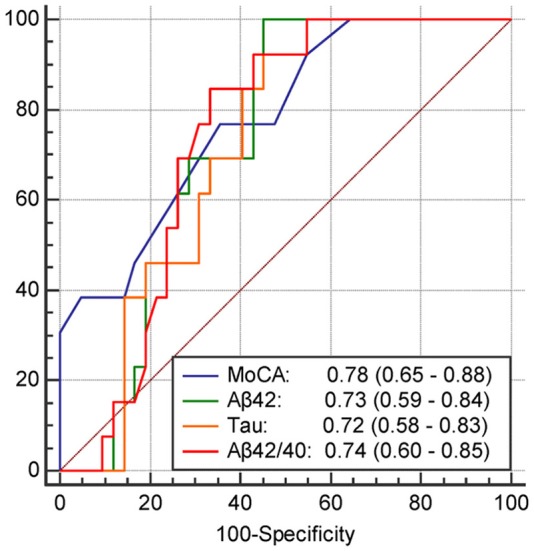
The receiver operating characteristics curve and the area under curves (95% confidence interval) of the variables predicting PSCI at 1 year. MoCA, The score of MoCA at 3 months; Aβ42, The plasma Aβ42 level at 3 months; tau, The plasma tau level at 3 months; Aβ42/40, The Aβ42/40 ratio at 3 months.

**Figure 4:**
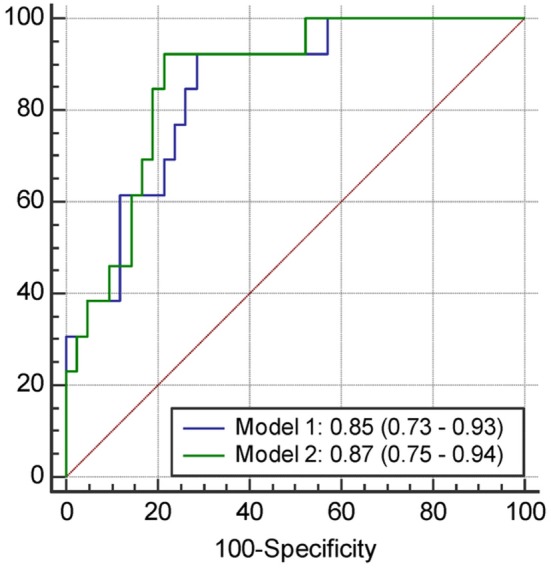
The receiver operating characteristics curve and the area under curves (95% confidence interval) of the variables predicting PSCI at 1 year. Model 1, Including the score of MoCA at 3 months and plasma Aβ42 level at 3 months; Model 2, Including the score of MoCA at 3 months and plasma tau level at 3 months.

The results of univariate and multivariate logistic regression analyses of variables predicting PSCI at 1 year are presented in Table 5. In univariate analysis, the MoCA score at 3 months, plasma Aβ42 and tau levels at 3 months, and Aβ42/40 ratio at 3 months were significant predictors, whereas the education level was a predictor with borderline significance (*P* = 0.092). Age, sex, vascular comorbidities, HbA1c, lesion volume on DWI, stroke lesion side, NIHSS score at any time point, Fazekas scale score, and plasma Aβ42 and tau levels within 7 days were not predictors of PSCI at 1 year. In multivariate analysis, plasma Aβ42, tau, and Aβ42/40 ratio were included in separate models because of their high association (correlation coefficient of plasma Aβ42 and tau within 7 days = 0.69, *P* < 0.001; correlation coefficient of plasma Aβ42 and tau at 3 months = 0.89, *P* < 0.001). MoCA scores at 3 months and the plasma Aβ42 level at 3 months as well as MoCA scores at 3 months and the plasma tau level at 3 months were included in the automatic forward variable selection models with good model fits (Nagelkerke *R*^2^ = 0.43 and 0.45, and Hosmer–Lemeshow test *P* = 0.55 and 0.52, respectively). However, the Aβ42/40 ratio at 3 months became non-significant after adjusting for MoCA scores at 3 months. Therefore, MoCA scores at 3 months and plasma Aβ42 and tau levels at 3 months were the most significant and independent predictors of PSCI at 1 year. The ROC curves of MoCA scores at 3 months, Aβ42 levels at 3 months, Aβ42/40 ratios at 3 months, and tau levels at 3 months for predicting PSCI at 1 year are displayed in Figure 3. In addition, the ROC curves of multivariate models to predict PSCI at 1 year are displayed in Figure 4.

**Table 5:** Univariate and multivariate logistic regression analyses of PSCI at 1 year.

**Characteristics (*n* = 55)**	**Crude OR (95% CI)**	***P*-value**	**Adjusted OR^†^ (95% CI)**	***P*-value**
Age	1.05 (0.98–1.13)	0.164		
Male sex	1.29 (0.24–7.02)	0.765		
Education level (year)	0.86 (0.72–1.02)	0.092		
Hypertension	0.70 (0.18–2.78)	0.616		
Diabetes mellitus	2.10 (0.60–7.40)	0.248		
Hemoglobin A1c (%) ≤ 7 days	1.02 (0.72–1.44)	0.921		
Hyperlipidemia	1.67 (0.39–7.04)	0.487		
NIHSS ≤ 7 days	0.97 (0.74–1.27)	0.810		
NIHSS at 1 year	1.12 (0.79–1.59)	0.513		
NIHSS at 3 months	1.44 (0.92−2.23)	0.101		
MoCA score at 3 months	0.77 (0.65–0.92)	0.005*		
DWI lesion volume (cm^3^)	1.06 (0.97–1.15)	0.181		
DWI lesion on the left side	1.47 (0.40–5.40)	0.562		
Fazekas scale (periventricular + deep white matter)	0.98 (0.62–1.55)	0.940		
Plasma biomarkers				
Amyloid beta-42 (pg/mL) ≤ 7 days	1.06 (0.83–1.35)	0.650		
Tau (pg/mL) ≤ 7 days	0.98 (0.91–1.05)	0.517		
Amyloid beta-42 (pg/mL) at 3 months	0.62 (0.42–0.92)	0.018*	0.63 (0.41–0.97)^†^	0.036*
Amyloid beta-40 (pg/mL) at 3 months	1.10 (0.98–1.21)	0.118		
Amyloid beta-42/40 ratio (%) at 3 months	0.88 (0.78–0.99)	0.032*	0.87 (0.76–1.00)^†^	0.055
Tau (pg/mL) at 3 months	0.87 (0.77–0.98)	0.024*	0.85 (0.73–0.99)^†^	0.031*

* *P < 0.05*,

† *The odds ratio of each plasma biomarker was separately adjusted for MoCA score at 3 months, DWI, diffusion-weighted image; MoCA, Montreal Cognitive Assessment; NIHSS, National Institute of Health Stroke Scale; PSCI, post-stroke cognitive impairment*.

## Discussion

The present study followed up 55 patients with acute ischemic stroke for 1 year to investigate the risk factors for and plasma biomarkers of PSCI. Although the average MoCA scores and the incidence of PSCI in all participants did not change between 3 months and 1 year, cognitive function improvements were observed in some patients, whereas cognitive function deterioration was observed in some patients. The patients with PSCI at 1 year had more prominent cognitive function decline than the patients without PSCI, which implied that a degenerative process occurred in the patients with PSCI at 1 year. Plasma Aβ42 and tau levels at 3 months were significant predictors of PSCI at 1 year, but not at 3 months, implying that ischemic-induced AD pathology may be one mechanism of PSCI development after 3 months of stroke.

The prevalence of PSCI in previous studies varied from 20% to 70% based on different definitions of PSCI and observational timing. Most studies have adopted cross-sectional designs and have evaluated patients at 3 months after a stroke (23–29), whereas some studies have applied longitudinal designs (30–33). In addition, commonly applied diagnostic tools include Diagnostic and Statistical Manual of Mental Disorders (DSM), Informant Questionnaire on Cognitive Decline in the Elderly (IQCODE), Mini-Mental State Examination (MMSE), or MoCA. In studies applying an MMSE score of <26 or an MoCA score <26 as the indicator of PSCI, the prevalence of PSCI was ~50% (23, 33). However, in studies applying an MMSE score of <24 as the indicator of PSCI, the prevalence ranged from 20 to 30% (27, 28, 30). In the present study, we selected an MoCA score <23 as the indicator of PSCI because an updated systemic review recommended the adoption of the MoCA score of 23 as the cutoff, considering its overall superior diagnostic accuracy and lower false positive rates compared with the initially recommended cutoff score of 26 (18). The PSCI prevalence in our study (13 of 55; 24%) is consistent with the results of other studies that have applied similar diagnostic criteria.

In the longitudinal studies, the PSCI prevalence remained relatively stable over months to 1 year, and improvements in cognitive function were observed in some patients, whereas cognitive function deterioration was observed in some patients (30, 31, 33). The findings are consistent with the results of our study: 7 of 13 patients with PSCI at 3 months recovered to a cognitively normal state at 1 year, and 7 of 42 cognitively normal patients at 3 months developed PSCI at 1 year.

Changes in cognitive function after a stroke occur at different stages, and it is critical to identify the risk factors for cognitive function deterioration. In the present study, a low education level and pre-existing white matter disease were the major risk factors for PSCI at 3 months; this finding is consistent with those of previous studies (28, 29). Age, diabetes mellitus, and stroke lesion volume were non-significantly associated with PSCI at 3 months in the present study; however, they have been reported as significant risk factors for PSCI in some studies (33, 34). Age and education level are not treatable risk factors, but vascular comorbidities are treated regularly in all patients with stroke; however, the incidence of PSCI remains high. Therefore, it is necessary to explore novel treatable risk factors.

Regarding the changes in MoCA scores from 3 months to 1 year, most patients with PSCI at 1 year (10 of 13) had MoCA scores that decreased by 2 points or more, and in most patients without PSCI at 1 year (39 of 42), their MoCA scores remained unchanged (score ± 1 point) or increased (by 2 points or more) (Table 3). Therefore, the development of PSCI from 3 months to 1 year could be a degenerative process. In patients with PSCI at 1 year, poor cognitive performance at 3 months is a recognized risk factor (30, 33), whereas the decrease in plasma Aβ42 or tau levels at 3 months has not been reported as a risk factor previously.

Decreased levels of plasma Aβ42 have been observed in patients with AD, which is consistent with increased Aβ42 deposition in the brain and a decreased Aβ42 level in the CSF (12, 13). The findings further suggest that Aβ42 deposition in the brain may cause PSCI development at 1 year. Animal studies have suggested that cerebral ischemia induces the deposition of Aβ42 (35, 36). In addition, Aβ42 deposition has been observed in one-third of patients with stroke and dementia using PET (37). Vascular risk factors increase the incidence of AD, and patients with AD are at higher risk of subsequent stroke (38). Therefore, AD pathology and ischemia are associated. However, it remains unclear whether AD pathology is linked to the onset of PSCI. In the present study, in the patients admitted within 7 days, plasma Aβ42 levels did not vary between patients with and without PSCI; however, low plasma Aβ42 levels at 3 months were associated with PSCI development at 1 year. Plasma Aβ42 levels exhibited a decreasing trend from 7 days to 3 months in the patients with PSCI at 1 year, although the decreases were non-significant (16.4 vs. 15.1, *P* = 0.102). Therefore, in the patients with PSCI in the present study, stroke potentially induced AD pathology, and AD pathology potentially did not exist before stroke occurrence.

The decreased plasma tau levels in the patients with PSCI in the present study may not be due to AD. In addition, plasma tau levels in patients with AD were higher than those in cognitively normal controls; however, the overlap in plasma tau levels between patients with AD and controls was high, and it has been reported that plasma tau levels partly reflect AD pathology (14). It has also been reported that plasma tau levels are positively associated with plasma Aβ42 levels, are not positively associated with CSF tau level levels, and are not different between patients with and without AD pathology (12). It is unclear whether the plasma tau levels are associated with the degree of taupathy in the brain. Therefore, in the present study, the decreased levels of plasma tau in the patients with PSCI could be explained by its association with the decreased plasma Aβ42 levels (12). Further investigations on the factors influencing plasma tau levels are required.

In the present study, the levels of plasma tau were more than 15 pg/mL on average; however, the average plasma tau levels measured using SIOMA have been reported to be ~4 pg/mL (39). The use of different assay technologies may result in inconsistency in the reported levels. In previous studies, the levels of plasma tau measured using IMR have been reported to be higher than 10 pg/mL (15, 40, 41). In the present study, the plasma tau levels obtained using IMR are higher than those obtained using SIMOA in other studies. The major reason may be that in SIMOA, magnetic beads are used to collect tau protein molecules and concentrate/purify low levels of the tau protein. The collected tau protein molecules are delivered to the wells, in which optical labels bind with the tau molecules to generate optical signals. During the process, the loss of tau protein molecules occurs. Conversely, in IMR, there is neither a concentration/purification step nor a delivery step, and the loss of tau protein molecules in IMR is lower than that in SIMOA. From a molecular structure perspective, two epitopes of the tau protein are required for SIMOA, whereas only one epitope is required for IMR. The tau protein is easily truncated. Although SIMOA cannot detect truncated tau protein molecules, IMR can do so. Therefore, IMR provides higher levels of the tau protein than SIMOA.

The present study had several limitations. First, the sample size was small, and a larger sample size is required to validate the results reported. Second, most patients in the present study had small-vessel disease with mild severity, which may influence the generalizability of the findings. However, large infarct caused by major artery occlusion or cardioembolism often causes PSCI instantly, which is not the target of most studies of PSCI. Third, we did not perform CSF analysis or PET, and plasma Aβ42 and tau levels are indirect measures of the condition of the central nervous system. However, it would be challenging to track changes in Aβ42 and tau levels based on CSF analysis or PET due to their invasiveness and high costs. Fourth, we used MoCA alone to diagnose PSCI. Multiple factors may influence the accuracy of a single neuropsychological test in the diagnosis of PSCI, including education level, physiological condition, and sensitivity or specificity of the test itself. The application of other evaluation methods in addition to MoCA, such as the NINDS-Canadian Stroke Network (NINDS-CSN) neuropsychological battery, could enhance the accuracy of the diagnosis.

## Conclusions

Changes in cognitive function occur at different stages after stroke. The pathogenesis of PSCI is complex. A low education level and pre-existing white matter disease are the key factors influencing PSCI development at 3 months, and ischemia-induced AD pathology is associated with PSCI development at 1 year. Such knowledge could facilitate the identification of patients at risk of developing PSCI. Exercise or cognitive training programs in addition to aggressive control of vascular risk factors may prevent PSCI in such patients.

## Data Availability

The datasets generated for this study are available on request to the corresponding author.

## Ethics Statement

The Institutional Review Board of Taipei Medical University approved this study. Written informed consent was obtained from all patients or their legal guardians.

## Author Contributions

N-FC, H-YC, and C-JH: conceptualization and methodology. N-FC and C-JH: data curation and writing–original draft. N-FC, S-PC, and C-JH: formal analysis. H-YC and C-JH: funding acquisition and supervision. N-FC, S-PC, L-KH, LC, and C-JH: investigation. Y-RC: project administration. S-PC, L-KH, LC, Y-RC, H-YC, and C-JH: resources. N-FC, S-PC, L-KH, LC, Y-RC, H-YC, and C-JH: writing–review and editing.

### Conflict of Interest Statement

The authors declare that the research was conducted in the absence of any commercial or financial relationships that could be construed as a potential conflict of interest.
